# An In Vitro Study to Evaluate the Antimicrobial Activity of a Zinc Citrate, Sodium Fluoride, Alum and Xylitol-Based Toothpaste Formulation

**DOI:** 10.7759/cureus.59413

**Published:** 2024-04-30

**Authors:** Kirti Shukla, Kranthi Kiran Pebbili, Seema V Bhagat, Kriti Kaushik, Arti P Sanghavi, Bhavesh P Kotak

**Affiliations:** 1 Medical Affairs, Dr. Reddy's Laboratories Ltd., Hyderabad, IND

**Keywords:** gum care, toothpaste, xylitol, sodium fluoride, zinc citrate, time-dependent antibacterial assessment, minimum bactericidal concentration, minimum inhibitory concentration, periodontitis

## Abstract

Introduction: Periodontitis is a prevalent condition significantly affecting oral health. Comorbid conditions, such as diabetes, can heighten the severity of periodontal disease and overall oral health. Therefore, to enhance oral health and manage comorbid conditions, comprehensive periodontal care is essential. This approach could involve using toothpaste containing antimicrobial ingredients in routine oral care. This paper presents the results of an in vitro study analysing the antimicrobial properties of the test formulation containing zinc citrate, alum, sodium fluoride, and xylitol-based toothpaste (Stolin-R). These ingredients work together to help in providing comprehensive oral care by controlling growth of bacteria majorly responsible for periodontal disease and thus maintaining optimal oral hygiene.

Aim: To determine the antimicrobial properties of zinc citrate, alum, sodium fluoride, and xylitol-based toothpaste formulation against key periodontal pathogens through in vitro analyses.

Materials and methods: The antimicrobial efficacy of test formulation is evaluated through minimum inhibitory concentration (MIC), minimum bactericidal concentration (MBC), and time-dependent antibacterial assessment against key periodontal pathogens, including *Porphyromonas gingivalis, Tannerella forsythia, Fusobacterium nucleatum, Prevotella intermedia, Streptococcus mutans*, and *Bacteroides fragilis*.

Results: The test formulation demonstrated potent antimicrobial effectiveness against *Bacteroides fragilis, Fusobacterium nucleatum, Porphyromonas gingivalis, Prevotella intermedia, Streptococcus mutans*, and *Tannerella forsythia*, by exhibiting low MIC and MBC. Additionally, significant bacterial reduction, exceeding 99.99%, was observed within five minutes, emphasising its potential as an effective adjunct in combating periodontal infection.

Conclusion: Zinc citrate, alum, sodium fluoride, and xylitol-based toothpaste formulation demonstrates significant antimicrobial activity against key periodontal pathogens, suggesting its potential as an effective agent for maintaining oral health and combating gingival infection.

## Introduction

Poor oral hygiene could lead to dental problems, including dental caries, periodontitis, and gingivitis, caused by germs and plaque accumulation [1]. Periodontitis emerged as a significant public health problem due to its high prevalence and potential consequences, such as tooth loss, disability, impaired chewing function, and compromised aesthetics. Periodontal and gingival diseases can also cause thinning of the tissues near the tooth, making them highly sensitive to heat and cold [2].

Bacterial activities on the tooth's surface cause gingival irritation and inflammation, and plaque accumulation at the gingival margin can lead to gingivitis, promoting further microbial proliferation through increased gingival crevicular fluid flow and blood supply [3]. In the progression of periodontitis, oxidative stress occurs in affected cells. Furthermore, affected cells produce the cytokines that trigger inflammation and activate the mechanisms that destroy tissues [4,5].

Dental professionals have long recognised the association between periodontitis and comorbid conditions. Epidemiological studies have revealed that comorbid conditions pose a significant risk factor for periodontitis, increasing the risk nearly three-fold compared to healthy individuals, particularly those with poor glycemic control. Individuals with comorbid conditions, such as diabetes tend to exhibit higher values for plaque, dental calculus, gingival inflammation, and deeper periodontal pockets, often requiring more extensive periodontal treatment and prophylactic procedures [6].

Research indicates that in addition to clinical factors related to comorbid conditions, the role of oral hygiene practices in preventing and controlling periodontal disease in patients with these conditions cannot be overlooked [7].

To manage gingival inflammation during periodontal therapy and maintenance, the use of adjunctive agents such as antimicrobial agents, astringents, and antiplaque substances has been suggested. Consequently, dentifrices, which often include adjunctive agents such as antimicrobial agents, astringents, and antiplaque substances, play a crucial role in daily oral hygiene maintenance [8]. In this context the significance of toothpaste as a product designed to maintain oral hygiene becomes paramount. It is crucial that oral care products are formulated to tackle the key microbially mediated diseases: caries, gingivitis, and periodontal diseases [9].

The Indian Society of Periodontology and the American Association of Periodontics recommend that maintaining oral hygiene is of utmost importance during and after periodontal therapy. Effective plaque control, crucial for oral health, can be achieved through regular toothbrushing. Additionally, the use of dentifrices containing active agents, can reduce plaque formation and the pathogenicity of periodontitis [10].

In the realm of oral health, various ingredients have been proposed for inclusion in toothpaste formulations due to their potential as antimicrobial agents. For example, zinc, alum, sodium fluoride, and xylitol, among others, are believed to contribute significantly to maintaining oral health. Each ingredient serves a specific purpose and enhances the overall effectiveness of the toothpaste [11,12].

Zinc salts have been advocated for their beneficial antimicrobial effects. Zinc inhibits microbial enzyme systems, including glycolytic enzymes in streptococci, making the organisms more sensitive to acid. In typical oral anaerobes like *Fusobacterium nucleatum* and *Prevotella intermedia*, zinc inhibits various enzymatic processes, including catabolism of amino acids, sugars, peptides, and oxidative metabolism. Additionally, zinc reduces halitosis by decreasing the concentrations of volatile sulphur compounds (VSCs) associated with halitosis and having direct and indirect inhibitory effects on anaerobe-mediated VSCs production. Zinc citrate and alum effectively control bacterial growth, ensuring optimal oral hygiene [11-16].

Sodium fluoride strengthens tooth enamel, offering protection against cavities. Topical fluoride in toothpaste has significantly reduced caries incidence in the last 30-40 years. Fluoride's primary mode of action against caries involves effects on enamel remineralisation. It also exhibits potentially caries-relevant antimicrobial effects, particularly on bacterial metabolism and glycolytic and acid-generating pathways. Xylitol inhibits bacterial growth by blocking their energy-making process due to xylitol 5-phosphate. It also causes problems by making glucose and xylitol compete for the same transporter, HPr-P, within the bacteria [17-19].

There are limited toothpaste formulations available that incorporate a combination of zinc citrate, alum, sodium fluoride, and xylitol. The amalgamation of these ingredients is believed to enhance the antimicrobial activity of the toothpaste. This, in turn, aids in the removal of bacteria that cause periodontitis, thereby potentially improving oral health outcomes.

This paper evaluates the antimicrobial testing of a novel toothpaste formulation containing 2% zinc citrate, 0.2% alum, 990 parts per million sodium fluoride, and 10% xylitol (Stolin-R). The combination of these four ingredients in Stolin-R is expected to work together, offering a comprehensive approach to oral care.

The study focused on assessing the antimicrobial efficacy of Stolin-R (active gum care) through in vitro experiments, explicitly determining the minimum inhibitory concentration, minimum bactericidal concentration, and time-dependent antibacterial efficacy of the toothpaste against various bacterial species associated with periodontal disease and tooth decay.

The bacterial species for the test were selected based on their role associated with oral health concerns such as periodontal diseases and tooth decay. *Porphyromonas gingivalis*, *Tannerella forsythia*, *Fusobacterium nucleatum*, *Bacteroides fragilis*, and *Prevotella intermedia* are commonly linked to periodontal diseases [16,20], while *Streptococcus mutans* contributes to tooth decay [21].

By exploring the antimicrobial action of this novel formulation in an in vitro setting, this study aims to provide valuable insights into a toothpaste formulation that can effectively target and combat the bacterial species associated with periodontal disease and dental plaque. This study could have substantial implications for improving oral health and providing support during active periodontal therapy.

## Materials and methods

minimum inhibitory concentration (MIC), minimum bactericidal concentration (MBC), and time-dependent kill rate studies were performed using a broth culture medium containing a bacterial suspension [22,23].

The media were used and incubated as specified in Table 1 throughout the study.

**Table 1:** Media and incubation conditions for microorganisms

Microorganism name	Media	Incubation condition	Incubation Period
Fusobacterium nucleatum	Tryptic soy medium with 5% defibrinated sheep blood agar	Anaerobically at 37°C	24-48 hours.
Bacteroides fragilis			
Prevotella intermedia			48-72 hours.
Porphyromonas gingivalis	Supplemented tryptic soy agar		
Streptococcus mutans	Mutans sanguis agar		
Tannerella forsythia	N-acetyl muramic acid medium agar		6 to 7 days.

Microbial culture

Microbial cultures were procured from the American Type Culture Collection. Approximately 3-10 x 10^5^ colony-forming unit (CFU)/mL concentration containing the microbial culture was used for the study [24]. The working culture was prepared to be twice the concentration of the final test, as there was a 1:2 dilution of the inoculum when combined with the test sample, so the final test concentration of the test culture was 5 x 10^5^.

Sample preparation

The preparation of the working solution involved dissolving a 10 gm sample in 10 mL of supplemented tryptic soy broth to create a stock solution with a concentration of 1000 mg/mL. Subsequently, a dilution series was established, resulting in final concentrations ranging from 1000 mg/mL to 3.91 mg/mL. The toothpaste was added at twice the final concentration in the study. This was necessary because there was a 1:2 dilution of the toothpaste when combined with the inoculum during the testing process. The concentrations tested for the toothpaste spanned from 500 mg/mL to 3.91 mg/mL, allowing for an assessment of its effectiveness in the broth dilution susceptibility tests.

Minimum inhibitory concentration

The micro broth dilution plate method determined each test microorganism's MIC values. Aseptically, sample and microbial culture were mixed for MIC determination in sterile tissue culture plates in triplicates, as shown in the below mentioned Table 2. Sample control, positive, and negative control were prepared as per Table 2 and Table 3, respectively; after MIC preparation, tissue culture plates were incubated in respective incubation conditions per Table 1. After the incubation, growth was checked for each well, and any turbidity observed was considered a sign of growth.

**Table 2:** Test and control preparation for MIC and MBC determination CFU = colony-forming unit; MIC = minimum inhibitory concentration; MBC = minimum bactericidal concentration

Sample concentration (mg/mL)	1000	500	250	125	62.5	31.25	15.63	7.81	3.91
Test: 1mL sample from each concentration was taken									
Control: 1mL sample from each concentration was taken									
Test: 1mL working inoculum (3 – 10 X 10^5^ CFU/mL) in each well separately									
Control: 1mL of supplemented tryptic soy broth were taken in each tube									
Test sample concentration (mg/mL)	500	250	125	62.5	31.25	15.63	7.81	3.91	1.95
Final test inoculum concentration: 1.5-5 X 10^5^ CFU/mL									

**Table 3:** Negative and positive control preparation for MIC and MBC determination CFU = colony-forming unit; MIC = minimum inhibitory concentration; MBC = minimum bactericidal concentration

Sample	Negative control	Positive control
Working Inoculum (3 – 10 X 10^5^ CFU/mL)	-	1mL
Supplemented tryptic soy broth	2mL	1mL
Test inoculum concentration	Nil	1.5-5 X 10^5^ CFU/mL

The MIC was determined as the lowest concentration of the sample dilution that inhibited the growth of the tested microorganism [25].

Minimum bactericidal concentration

MBC was determined after determining the MIC. After incubation, samples from the MIC wells are taken and subcultured on solid agar plates by direct streaking. The selection of solid media and incubation conditions for each microorganism was as per Table 1. Control wells were also subcultured on solid agar media. After incubation, the plates were examined for bacterial growth. The lowest concentration of toothpaste that completely prevents bacterial growth on the solid medium was defined as the MBC [12,26].

Time-dependent antibacterial efficacy

This test was performed individually for each test microorganism. In a tube, 5mL of standardised working inoculum (approximately 1.5-5 X 10^5^CFU/mL) and 5mL of MBC concentration of the sample were taken. The mixture was vortexed thoroughly, and the tubes were anaerobically incubated at 37°C. Bacterial enumeration was conducted on 1mL of the test mixture at various time intervals (i.e., one minute, two minutes, five minutes, 10 minutes, one hour, six hours, 12 hours, and 24 hours) by spread plate method in duplicate [15]. The plates were incubated per the microorganism's incubation conditions in Table 1. After incubation, the plates were examined for colony count. The colony count was multiplied by the appropriate dilution factor to calculate the final inoculum size, and log reduction was calculated from the initial and final count of inoculum [27].

## Results

The MIC values were determined to evaluate Stolin-R toothpaste's antimicrobial effectiveness against diverse periodontal pathogens. For *Bacteroides fragilis*, the MIC was measured at 3.91 mg/mL, indicating the concentration required to inhibit bacterial growth. *Fusobacterium nucleatum* exhibited an MIC of 7.81 mg/mL, while *Porphyromonas gingivalis* displayed an MIC of 3.91 mg/mL. Similarly, *Streptococcus mutans*, *Prevotella intermedia*, and *Tannerella forsythia* demonstrated an MIC of 7.81 mg/mL. These findings provide insights into the toothpaste's capacity to impede the growth of specific periodontal pathogens, laying the foundation for understanding its potential role in oral care (Table 4).

**Table 4:** MIC of Stolin R toothpaste against periodontal pathogens S = Sensitive; R = Resistance; MIC = minimum inhibitory concentration

Microorganisms	Toothpaste concentration (mg/mL)								
	500	250	125	62.5	31.25	15.63	7.81	3.91	1.95
Bacteroides fragilis	S	S	S	S	S	S	S	S	R
Fusobacterium nucleatum	S	S	S	S	S	S	S	R	R
Porphyromonas gingivalis	S	S	S	S	S	S	S	S	R
Prevotella intermedia	S	S	S	S	S	S	S	R	R
Streptococcus mutans	S	S	S	S	S	S	S	R	R
Tannerella forsythia	S	S	S	S	S	S	S	R	R

The investigation into the antimicrobial efficacy of Stolin-R toothpaste extended to determining the MBC value against various periodontal pathogens. The MBC values indicate the concentration required for complete eradication of bacterial growth. For *Bacteroides fragilis*, the MBC was 15.63 mg/mL, while *Fusobacterium nucleatum* displayed an MBC of 31.25 mg/mL. *Porphyromonas gingivalis *exhibited an MBC of 7.81 mg/mL, and both *Prevotella intermedia* and *Streptococcus mutans* demonstrated an MBC of 31.25 mg/mL. Similarly, *Tannerella forsythia* presented an MBC of 31.25 mg/mL. These results underscore the toothpaste's potential to achieve bactericidal effects against specific periodontal pathogens, contributing valuable insights into its antimicrobial properties in oral care (Table 5).

**Table 5:** MBC of Stolin R toothpaste against periodontal pathogens NG = No Growth; G = Growth; MBC = minimum bactericidal concentration

Microorganisms	Toothpaste concentration (mg/mL)								
	500	250	125	62.5	31.25	15.63	7.81	3.91	1.95
Bacteroides fragilis	NG	NG	NG	NG	NG	NG	G	G	G
Fusobacterium nucleatum	NG	NG	NG	NG	NG	G	G	G	G
Porphyromonas gingivalis	NG	NG	NG	NG	NG	NG	NG	G	G
Prevotella intermedia	NG	NG	NG	NG	NG	G	G	G	G
Streptococcus mutans	NG	NG	NG	NG	NG	NG	G	G	G
Tannerella forsythia	NG	NG	NG	NG	NG	G	G	G	G

**Figure 1:**
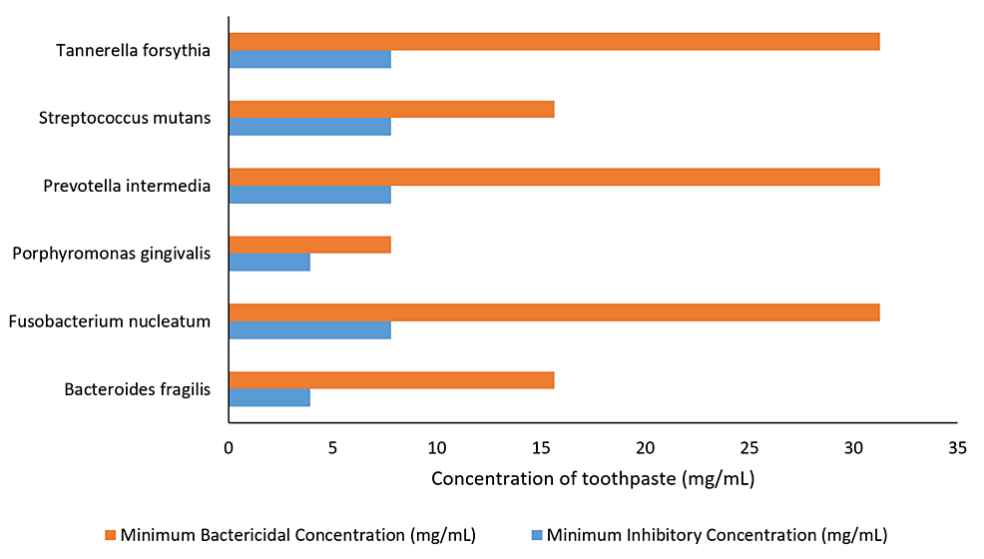
MIC and MBC of Stolin R against periodontal pathogens MIC = minimum inhibitory concentration, MBC = minimum bactericidal concentration

In a comparative analysis, *Bacteroides fragilis* demonstrated the lowest MIC at 3.91 mg/mL, while *Porphyromonas gingivalis* had a comparable MIC of 3.91 mg/mL. *Fusobacterium nucleatum*, *Prevotella intermedia*, and *Tannerella forsythia* exhibited slightly higher MIC values at 7.81 mg/mL. For MBC, *Porphyromonas gingivalis* displayed the lowest value at 7.81 mg/mL, followed closely by *Bacteroides fragilis* and *Streptococcus mutans*. The results suggest variations in susceptibility among the tested microorganisms (Figure 1).

**Figure 2:**
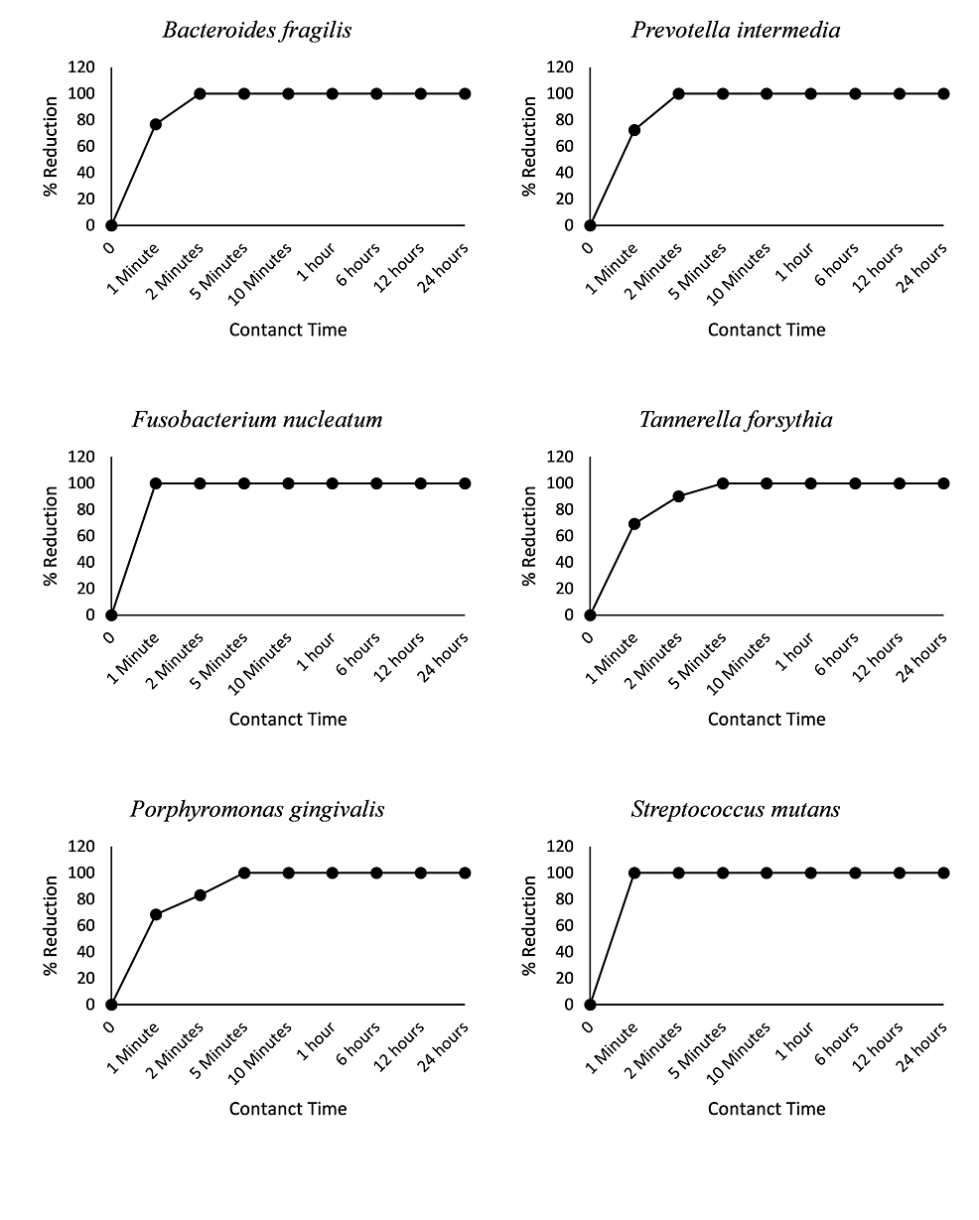
Time-dependent kill rate of Stolin R against periodontal pathogens

The time-dependent antibacterial assessment underscores Stolin-R toothpaste's swift and robust antibacterial efficacy against various periodontal pathogens. Results show that *Fusobacterium nucleatum* demonstrated a reduction of over 99.99% in just one minute. *Bacteroides fragilis*, *Prevotella intermedia* and *Streptococcus mutans* followed closely, with reduction of more than 99.99% after two minutes of exposure. After five minutes, *Porphyromonas gingivalis* and *Tannerella forsythia* showed a significant reduction, exceeding 99.99% (Figure 2). These results highlight the rapid and potent antibacterial action of the toothpaste.

These results collectively emphasise the quick and potent time-dependent antibacterial effects of Stolin-R toothpaste, suggesting its efficacy in rapidly reducing bacterial loads during routine oral care practices.

## Discussion

The study investigated the antimicrobial properties of the test formulation, Stolin R. Through assessments of MIC and MBC, it was observed that Stolin R effectively combats microbial growth. Furthermore, a time kill study revealed rapid and efficient eradication of test microorganisms within a short span of time. The pathogens targeted in this study included *Bacteroides fragilis*, *Fusobacterium nucleatum*, *Porphyromonas gingivalis*, *Prevotella intermedia*, *Streptococcus mutans*, and *Tannerella forsythia*. These results highlight the potential of Stolin R as an effective antimicrobial agent.

The MIC plays a pivotal role in evaluating the effectiveness of antimicrobial agents. It signifies the lowest concentration of a substance necessary to inhibit visible growth of microorganisms. The MBC represents the minimum concentration of an antimicrobial agent required to kill bacteria, rather than merely inhibiting their growth. In the context of periodontal health, this finding holds significant implications [14].

In this study, a test formulation containing zinc citrate, alum, NaF, and xylitol (Stolin-R) showed MIC values ranging from 3.91 mg/mL to 7.81 mg/mL and also demonstrated a low MBC, with concentrations ranging from 7.81 mg/mL to 31.25 mg/mL against periodontal pathogens.

MIC and MBC results align with the study that investigated toothpaste containing similar types of ingredients [8,12-16,19].

Furthermore, the rapid and significant reductions demonstrated in the time-dependent antibacterial assessment, ranging from one to five minutes, align with studies emphasising the importance of quick bacterial reduction in oral care [13,14,28].

The results demonstrate that the test formulation’s ingredients effectively inhibit the microbial growth of periodontal pathogens. Numerous studies have also highlighted the efficacy of zinc, alum, sodium fluoride, and xylitol-like compounds against periodontal microorganisms, including both gram-positive and gram-negative species.

Specifically, zinc ions and fluoride interfere with essential microbial enzyme systems, disrupting their function [12,13]. Notably, zinc and fluoride exhibit a broad-spectrum activity against periodontal pathogens [13,28].

Research shows that glutamate dehydrogenase and 2-oxoglutarate reductase play crucial roles in the metabolic pathways of periodontal microorganisms. Zinc (Zn) acts as an inhibitor for these two enzymes by binding to a site on the sulfhydryl group of the enzyme. Additionally, Zn has antioxidant effects, inhibiting respiration and inducing H_2_O_2_ production. H_2_O_2_ is considered toxic for streptococcal and enterococcal infections, as it enhances peroxidase killing [14].

Pizzey et al. conducted a study demonstrating that formulations containing zinc exhibit bactericidal properties against several oral pathogens, including *Streptococcus mutans*, *Fusobacterium nucleatum*, *Prevotella intermedia*, and *Porphyromonas gingivalis* [12]. These findings highlight the potential of zinc-based compounds in combating oral infections.

In another study, Lavaee et al. investigated the effects of zinc and fluoride-containing formulations. They observed inhibitory efficacy against both gram-positive and gram-negative microorganisms, including *Streptococcus mutans* and *Fusobacterium nucleatum* [13]. This suggests that combining zinc and fluoride could enhance the antimicrobial properties of oral products.

Furthermore, Sheng et al. reported that zinc ions act as potent inhibitors. Their research demonstrated that test formulations containing zinc ions effectively kill periodontal microorganisms, such as *Fusobacterium nucleatum* and *Prevotella intermedia* [14]. These findings emphasize the role of zinc in maintaining oral health.

Marquis reported that fluoride directly acts as an enzyme inhibitor for microorganisms. For example, it inhibits the enolase glycolytic enzyme. Fluoride ions also inhibit the proton-translocating activity of F-ATPases and are thought to act by mimicking phosphate, forming complexes with ADP at the reaction centres of the enzymes [18].

Buzalaf et al. reported that fluoride-containing toothpaste reduces oral bacteria in the teeth, saliva, and multiple soft tissue locations [19].

Typically, adults use 0.52gm to 1.19gm of toothpaste during a single toothbrushing session [29]. The toothpaste undergoes a four-fold dilution with saliva in the mouth during brushing [30], resulting in an approximate concentration of 0.13 gm/mL (130,000 PPM) to 0.30 gm/mL (300,000 PPM) of toothpaste coming into contact with the mouth. The study revealed that Stolin R toothpaste, at a concentration of 31.25 PPM, effectively inhibited all tested microorganisms during in vitro testing, indicating its high efficiency.

This highlights the toothpaste’s remarkable effectiveness in promoting oral hygiene and indicates its potential as an efficient gum care product. While the in vitro findings are promising, it’s important to recognize the inherent limitations of such studies. In vitro experiments take place in a controlled laboratory environment, this artificial setting may not fully replicate the intricate dynamics of the oral cavity as it exists in real-world scenarios. The complexities of the oral environment include factors like saliva composition, microbial interactions, and the influence of host immune responses. These elements play a crucial role in maintaining oral health and preventing diseases. Unfortunately, in vitro studies cannot fully capture these dynamic interactions. Therefore, further clinical research is necessary to validate the promising results observed [28].

## Conclusions

Periodontal problems are primarily caused by bacteria, leading to issues such as plaque, inflammation, and oxidative stress. Experimental results demonstrate that the Stolin-R formulation exhibits significant MIC and MBC values against bacteria within five minutes of contact time. These findings provide valuable insights into the potential of Stolin-R as a promising oral care agent that offers relief from periodontal pathogens and potentially revolutionizes oral care. Given these promising results, it is recommended to conduct clinical validation of Stolin-R. Furthermore, long-term safety assessments of this formulation are crucial to ensure its practical application in oral care.
